# Piezoelectric Accelerator

**DOI:** 10.1038/s41598-018-34831-8

**Published:** 2018-11-07

**Authors:** O. O. Ivashchuk, A. V. Shchagin, A. S. Kubankin, I. S. Nikulin, A. N. Oleinik, V. S. Miroshnik, V. I. Volkov

**Affiliations:** 1 Belgorod National Research University, Belgorod, Russia; 2 Kharkov Institute of Physics and Technology, Kharkov, Ukraine; 3 P.N. Lebedev Physical Institute of the Russian Academy of Sciences, Moscow, Russia; 4 John Adams Institute at Royal Holloway, University of London, Egham, UK

## Abstract

Here we propose the conception of small-size piezoelectric accelerator of charged particles that operates due to the piezoelectric effect at varying mechanical force applied to piezoelectrics in vacuum. The accelerating voltage and the energy of accelerated particles are estimated. In the proof-of-principle experiment we demonstrate the effect of the emission of X-ray radiation at the mechanical compression of piezoelectric ceramics in vacuum. The compression leads to the appearance of charges and potentials on the surfaces of the piezoelectrics and also to the arising of the electric field in vacuum. Electrons are accelerated in the electric field, strike the matter and produce the X-ray radiation. In the experiment, we have observed emission of the characteristic and bremsstrahlung X-ray radiation of energy up to 60 keV due to the compression of piezoelectric ceramics in vacuum. This means that electrons are accelerated in the piezoelectric accelerator up to the energy at least of 60 keV. The agreement of calculated and experimental data confirms the conception. Advantages of the piezoelectric accelerator and possibilities of its development and applications are discussed.

## Introduction

Usually, elementary particles and ions are accelerated by one of the classic methods of acceleration^1^. But in more recent times, a few new methods of particle acceleration up to the energy of about 100 keV in small-size accelerators without any outer high-voltage power supply have been proposed. For instance, X-ray radiation is produced by electrons accelerated in the electric field arising in vacuum due to pyroelectric^2–4^ and triboelectric^5,6^ effects or in the high-frequency field which is generated by a piezoelectric transformer in vacuum^7–9^. Here we propose the piezoelectric accelerator which operates due to piezoelectric effect at varying the mechanical force applied to piezoelectrics in vacuum.

Piezoelectric ceramics is widely used in gas igniters for production of high voltage and sparks. A spark discharger in a piezoelectric gas igniter restricts voltage that could be obtained due to piezoelectric effect. However, much higher voltage can be obtained if the piezoelectrics are installed and compressed in vacuum without a discharger.

### Conception of piezoelectric accelerator

Let us estimate the voltage which can be obtained at the compression of piezoelectrics in vacuum. Consider a layer of piezoelectric ceramic of thickness *h* polarized in the direction perpendicular to the layer surfaces. The layer is electrically isolated and the compression provides mechanical strength *P* in the ceramics. The potential difference or voltage *V* between the surfaces of the layer is

1 $$V=\frac{Q}{C},$$

where *Q* is the surface charge per unit of area which arises due to the piezoelectric effect,

2 $${Q}={d}_{33}\cdot P,$$

d_33_ is the piezoelectric modulus, *C* is the capacity per unit of area,

3 $$C=\frac{\varepsilon \cdot {\varepsilon }_{0}}{h},$$

${\varepsilon }_{0}=8.85\cdot {10}^{-12}\frac{F}{m}$ is the dielectric permittivity of free space, *ε* = *ε*_33_/*ε*_0_ is the relative dielectric permittivity of the piezoelectric ceramics along the polarization vector. As a result, we obtain voltage

4 $$V=\frac{{d}_{33}\cdot P\cdot h}{\varepsilon \cdot {\varepsilon }_{0}},$$

which is proportional to the pressure and the layer thickness. For example, the estimation with use of values ${d}_{33}=1.7\cdot {10}^{-10}\frac{C}{N}$, *ε* = 1300, *h* = 10 cm and maximum mechanical strength ${P}_{\max }=3.35\cdot {10}^{8}\frac{N}{{m}^{2}}$ (see properties of the ceramics in the Supplementary info online) gives maximal voltage *V* = 0.5 MV at the electric field strength along the edges of the layer $\frac{V}{h}=50\,\frac{kV}{cm}$.

The leakage current can lead to some reduction of the estimated voltage. However, even some reduced voltage of about hundred(s) kV could be interesting for acceleration of charged particles in a small-size accelerator. In order to avoid an additional electric isolation, one can use the assembly of two ceramics layers with opposite directions of the polarization vectors. In this case, the outer surfaces can be grounded and high voltage will be produced in the inner surfaces of the layers at compression of the both layers.

A layer of conductive material (high-voltage electrode) can be inserted between the inner surfaces of the piezoelectric layers. The voltage *V* at the electrode is the same (1) in such assembly, but the charge (2) and the capacity (3) are doubled^10^. The high voltage at the electrode can be used as accelerating voltage in the accelerator. Elementary particles or ions can be accelerated in such accelerator up to energy |*q*|*V*, where *q* is the charge of the particles or ions. Thus, the simple estimation shows that elementary particles and ions can be accelerated up to energies of hundred(s) keV by accelerating voltage produced due to the piezoelectric effect at compression of piezoelectrics in vacuum.

### Proof-of-principle experiment

**Figure 1:**
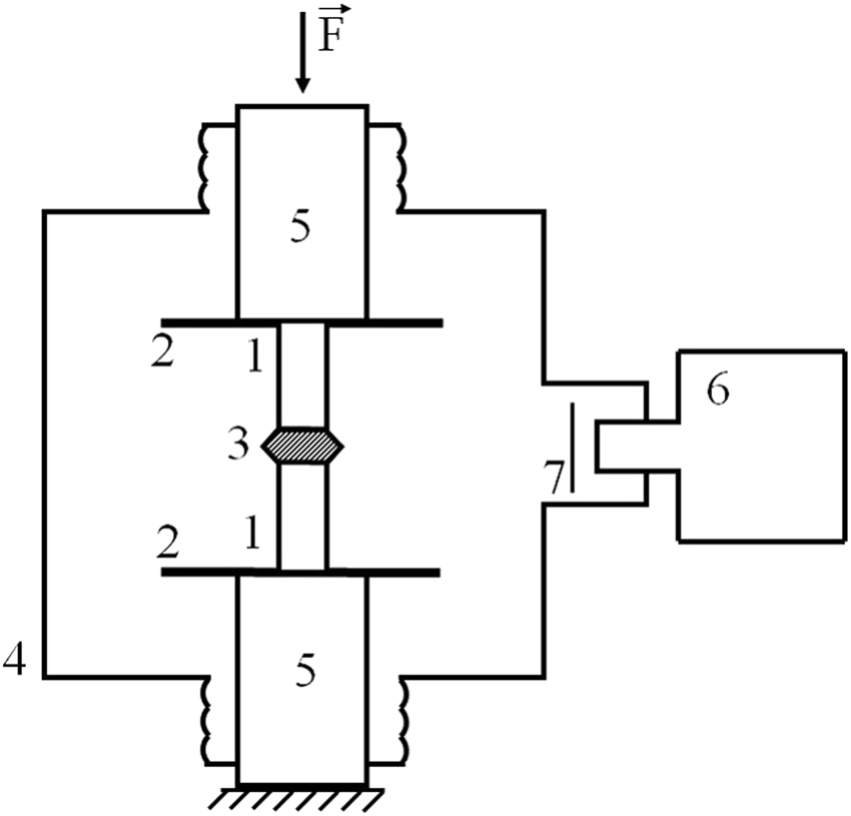
The scheme of the experimental setup. The cylindrical assembly of two piezoelectric ceramic cylinders 1 with copper high-voltage electrode 3 is installed in vacuum chamber 4 between two metal grounded disks 2. The variable force $\overrightarrow{F}$ can be applied along the assembly axis through metal cylinders 5. The spectra of X-ray radiation are measured with X-ray detector 6. Mylar foil 7 is installed before the entrance window of the detector.

In order to verify above described conception of the piezoelectric accelerator, we performed experimental research for acceleration of electrons and observation of X-rays at compression of small pieces of piezoelectric ceramics in vacuum. The experiments have been performed in the Belgorod National Research University, Russia. The scheme of the experimental setup is shown in Fig. 1. The cylindrically-symmetric assembly consisting of two piezoelectric ceramics cylinders and the high-voltage electrode is installed in the vacuum chamber between two grounded zinc-plated iron disks of 50 mm in diameter. The copper high-voltage electrode is inserted between the two ceramic cylinders. The electrode height is 9.0 mm, the maximum diameter is 18.8 mm and the diameter of the both electrode bases is 10.4 mm. The vacuum chamber of 145 mm in diameter and of 103 mm in height is produced from stainless steel. The controlled pressing force$\overrightarrow{F}$ is produced by dual column load frame machine Instron 3369. One can see photographic images of the experimental setup in the Supplementary Fig. S1 and inner view of the vacuum chamber in the Supplementary Fig. S2. The ceramics is polarized along the cylinder axis. Two ceramic cylinders in the assembly have opposite directions of the polarization vectors to produce high voltage at the electrode installed between the cylinders.

Lead barium zirconate titanate ferroelectric ceramics is used as piezoelectric elements in the experiments. The ceramics elements are produced in the form of cylinders having 6.4 mm diameter and 15 mm length to be applied in the common piezoelectric kitchen gas igniters. The same ferroelectric ceramic cylinders were used before in our experiments with a pyroelectric accelerator^4^. One can see properties of the ceramic cylinders in the Supplementary info online.

The spectra of X-ray radiation were measured by the X-ray spectrometer consisting of 0.5 mm thick Si detector Amptek XR-100SDD with collimated area 17 mm^2^ and digital pulse processor PX5 connected to a laptop. The 12 μm thick beryllium entrance window of the detector is sunk into the vacuum chamber. The distance between the window and the assembly axis is 82 mm. The 110 μm thick mylar foil is placed before the detector to prevent a damage of the entrance beryllium window in the case of piezoelectric ceramics fracture. The peaking time of the X-ray spectrometer is 1.0 μs. In our measurements, the energy resolution of the spectrometer (FWHM) was 240 eV at 8.0 keV Cu K_α_ peak. The registration efficiency of the spectrometer slows down at X-ray energy below ~5 keV, mainly because of the attenuation of X-rays in the mylar foil, and at energy above ~10 keV because the transparency of the 0.5 mm thick Si detector increases for harder X-rays.

The measurements of the spectra were carried out at the pressure of the residual gas (air) in the chamber about 0.1 mTorr at room temperature. The spectra of X-ray radiation were measured at compressing the assembly with two opposite alignments of the polarization vectors of the piezoelectric cylinders. In the one case, the polarization vectors of the both cylinders are directed toward the high-voltage electrode. In the other case, the polarization vectors of the both cylinders are directed from the high voltage electrode. Thus, the piezoelectric cylinders were connected mechanically in series and electrically in parallel (as well as in a gas igniter) in the both our measurements.

**Figure 2:**
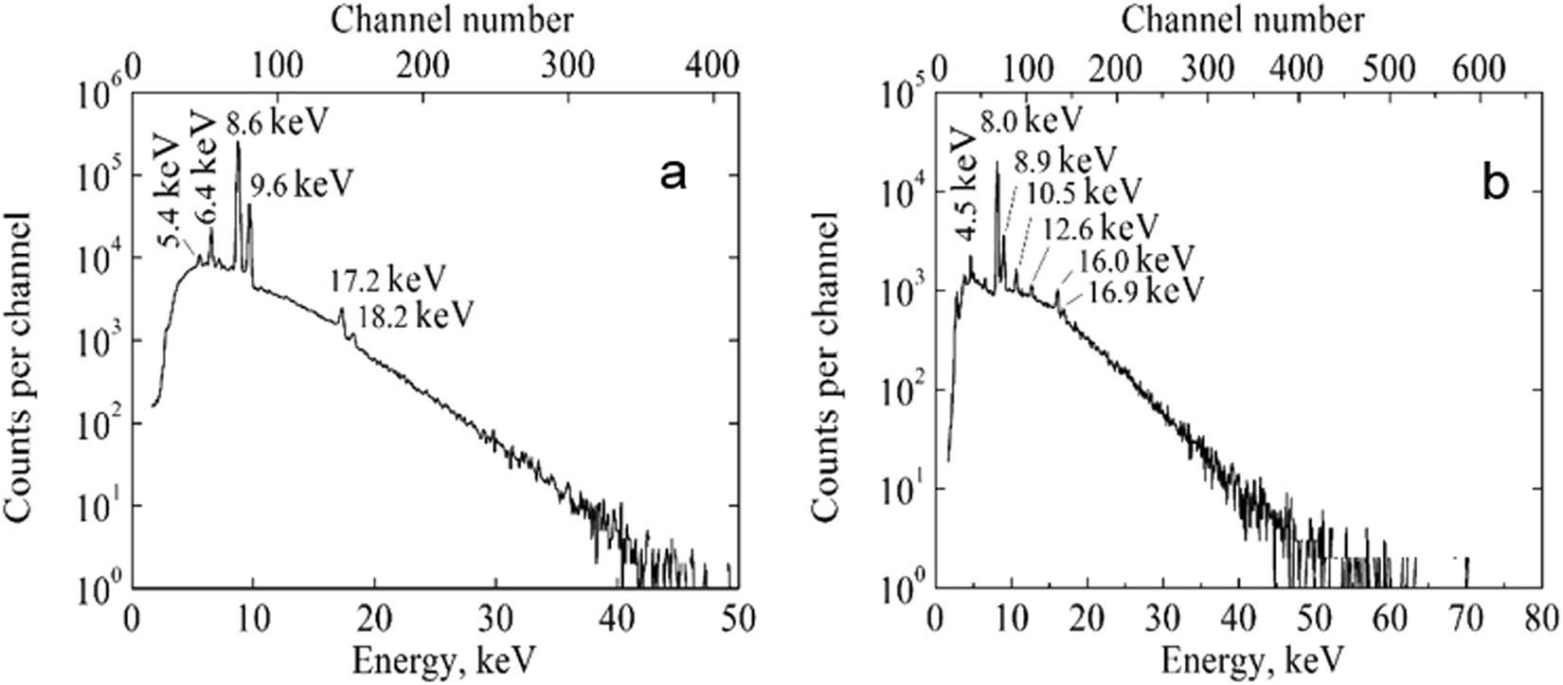
The spectra of X-ray radiation measured at a single compression of the assembly without corrections for registration efficiency of the spectrometer. Spectra were measured at the negative (**a**) and positive (**b**) potentials at the high-voltage electrode.

In the first measurement, the negative potential is produced at the high-voltage electrode due to the piezoelectric effect during the compression of the assembly. The duration of the compression up to the maximum force of 5.5 kN $(P=1.71\cdot {10}^{8}\frac{N}{{m}^{2}})$ was 3 seconds. Electrons accelerate in the electric field from the high-voltage electrode toward the metal disks and walls of the chamber and strike them. The X-ray spectrum presented in Fig. 2a is measured during the compression and for 139 seconds after fixing the maximum force. The spectrum contains the set of peaks of characteristic X-ray radiation against the smooth background of the bremsstrahlung. The spectral peaks at energies of 5.4 keV and 6.4 keV correspond to the K_α_ lines of Cr and Fe atoms, respectively, of the stainless steel of the chamber. The spectral peaks at energies of 8.6 keV and 9.6 keV correspond to the K_α_ and K_β_ lines of Zn atoms, respectively, of the zinc coating of the metal disks. The spectral peak at energy of 17.2 keV is due to the simultaneous registration of two coinciding K_α_ Zn photons. The spectral peak at energy of 18.2 keV is due to the registration of coinciding K_α_ and K_β_ Zn photons. The total number of registered counts in the spectrum is 1.24 · 10^6^. The maximum energy of the bremsstrahlung reaches nearly 45 keV.

Before the second measurement, the both ceramic cylinders were reversed and the positive potential was produced at the high-voltage electrode of the assembly at the compression. The duration of the compression up to the maximum force 8.6 kN $(P=2.67\cdot {10}^{8}\frac{N}{{m}^{2}})$ was 4 seconds. Electrons accelerate in the electric field toward the high-voltage copper electrode and ceramics and strike them. The X-ray spectrum measured during the compression and for 41 seconds after fixing the maximum force is presented in Fig. 2b. The spectrum contains another set of peaks of characteristic X-ray radiation against the smooth background of the bremsstrahlung. The energies of the peaks in this set are different from previous ones. The spectral peaks of energies 8.0 keV and 8.9 keV correspond to the K_α_ and K_β_ lines of Cu atoms, respectively, of the high-voltage electrode. The spectral peak at energy of 16.0 keV is due to the registration of the two coinciding K_α_ Cu photons. The spectral peak at energy of 16.9 keV is due to the registration of coinciding K_α_ and K_β_ Cu photons too. Other most prominent peaks are due to the characteristic X-ray radiation of ceramics atoms. The peak at energy of 4.5 keV corresponds to the K_α_ lines of the Ti and the L_α_ lines of the Ba. The peaks of energies 10.5, 12.6 keV are due to the L_α_, L_β_ lines of Pb, respectively. The spectral peaks due to K lines from the atoms Zr, Ba and Pb are not seen because of restricted energy of the accelerated electrons and/or a low radiation yield. The total number of registered counts in the spectrum is 1.90 · 10^5^. The maximum energy of the bremsstrahlung reaches of 60 keV.

In the both our measurements, the maximum X-ray energy and the X-ray yield on average decreased in time after fixing of the maximum force because of the high-voltage electrode discharging during about minute. But the appearance of spectral peaks at energies equal to the sum of energies of K-lines of Zn and Cu means that in the beginning of the both measurements the current of accelerated electrons, and the X-ray flux, and the counting rate of the spectrometer sufficiently exceed the average ones that leads to registration of coinciding photons due to the pile up effect.

The charge of the opposite sign appears at the high-voltage electrode after the discharge of the electrode and following reduction of the applied force to zero. As a result, electrons accelerate in opposite direction and we observed the X-ray spectra with another set of peaks of characteristic X-rays in both cases (see Fig. 2a,b). The more detail research of the temporary dependencies of the X-ray spectra will be published elsewhere.

## Results and Discussion

The emission of X-rays is the clear evidence of acceleration of electrons. The maximum energy of accelerated electrons should exceed the maximum energy of emitted bremsstrahlung. Therefore, we conclude that maximum energy of accelerated electrons is no less than 45 keV and the potential at the high-voltage electrode is no more than −45 kV in the first measurement (Fig. 2a) and the electron energy is no less than 60 keV and the potential is no less than +60 kV in the second measurement (Fig. 2b). The maximum electric field strength along every ceramic cylinder is 30 kV/cm in the first measurement and 40 kV/cm in the second one. Further increasing of the force leads to the ceramic’s fracture or to spark discharges in our experiments.

Let us compare experimentally observed accelerating voltage to the estimated by formula (4) potentials. In the estimation, we used properties of the ceramics listed in the Supplementary info online at the assumption that *ε* increases linearly at the increasing of the mechanical strength and voltage,

5 $$\varepsilon =\frac{{\varepsilon }_{{\rm{\max }}}-{\varepsilon }_{{\rm{\min }}}}{{P}_{{\rm{\max }}}}P+{\varepsilon }_{{\rm{\min }}},$$

where ${\varepsilon }_{\min }=600$, ${\varepsilon }_{\max }=1300$, ${P}_{\max }=3.35\cdot {10}^{8}\frac{N}{{m}^{2}}$ according to the ceramics producer’s specification given in the Supplementary info online.

The negative potential calculated by formulae (4,5) at ${d}_{33}=1.7\cdot {10}^{-10}\frac{C}{N}$, $\varepsilon =957$, $h=1.5$ cm, $P=1.71\cdot {10}^{8}\frac{N}{{m}^{2}}$ is 51.5 kV that is close to the experimental value 45 kV. The positive potential calculated by the same formulae at ${d}_{33}=1.7\cdot {10}^{-10}\frac{C}{N}$, $\varepsilon =1158$, $h=1.5$ cm, $P=2.67\cdot {10}^{8}\frac{N}{{m}^{2}}$ is 66.4 kV that is close to the experimental value 60 kV. The both estimated voltages exceed the experimental data for about 11–15%. The exceeding may be due to some current leakage, the edge effects, and the capacity of the high-voltage electrode that were no taken into account in our simple estimation. The qualitative agreement between the estimated and measured accelerating voltages confirms the validity of the conception of the piezoelectric accelerator.

Note, that the measured values of the maximum energy of the bremsstrahlung and corresponding accelerating voltage *V* can be used for direct experimental determination of the value *ε* of a piezoelectric at high mechanical strength *P* and voltage *V*. The value of *ε* can be found from Eq. (4),

6 $$\varepsilon =\frac{{d}_{33}\cdot P\cdot h}{V\cdot {\varepsilon }_{0}}.$$

Let us compare surface charges produced in piezoelectric and pyroelectric accelerators. Eqs. (1,3) are valid for both piezoelectric and pyroelectric effects. Therefore, one can find from these Eqs. the relation *A* of the surface charges produced by piezoelectric and pyroelectric elements of equal thicknesses at the same voltages

7 $$A=\frac{{Q}_{piezo}}{{Q}_{pyro}}=\frac{{\varepsilon }_{piezo}}{{\varepsilon }_{pyro}},$$

where ${Q}_{piezo}$, ${\varepsilon }_{piezo}$ and ${Q}_{pyro}$, ${\varepsilon }_{pyro}$ are the surface charge and the permittivity of a piezoelectric and pyroelectric, respectively. In present experiment, we used ceramics with ${\varepsilon }_{piezo} \sim 1000$ that is typical value for piezoelectric ceramics. In pyroelectric accelerators, pyroelectric crystals like *LiNbO*_3_ and *LiTaO*_3_ are often applied (see, e.g.^2–4,11,12^). Their *ε*_*pyro*_ are equal to 31 and 44, respectively. It appears that relation $A\gg 1$ and the charge produced in piezoelectric accelerator can in tens times exceed one produced in a crystalline pyroelectric accelerator at the same voltage. (However, the relation *A* may be not so significant for pyroelectric accelerator built on pyroelectric ceramics^4^). Besides, the application of the assembly of two piezoelectrics with high voltage electrode doubles the charge at the same voltage. The increasing of the charge allows proportionally increase the current and power of accelerated beam.

Compare also velocities of the charge production. In a piezoelectric accelerator, the full charge can produced for a few seconds at the compression. In typical pyroelectric accelerator, the full charge can produced for about 10 minutes at heating of a pyroelectric for about 100°K^11^. Thus, the velocity of the charge production in piezoelectric accelerator can exceed one in a pyroelectric accelerator for about two orders of magnitude.

The small-size piezoelectric accelerator seems is envisaging further development due to above described advantages.

Let us discuss some possibilities for further development of piezoelectric accelerator. First of all, draw attention to the radial-like motion of accelerating electrons around of the high-voltage electrode and the ceramic cylinders realized in the proof-of-principle experiments. In future experiments, the electron and X-ray fluxes can be increased by providing the directed electron beam instead of the radial-like motion of electrons. Besides, periodical variation of the value of the force applied to piezoelectrics can be used for increasing of the fluxes. In particular, periodical impacts can be used for production of pulsed accelerating high voltage and the pulsed beam of particles. The ceramics with maximum values of the relation $\frac{{d}_{33}}{{\varepsilon }_{33}^{T}}$ and of the limit of the mechanical strength is the most suitable for obtaining of high accelerating voltage in a piezoelectric accelerator.

The piezoelectric accelerator can be used in order to accelerate electron and ion beams, to produce increased number of neutrons due to (d,n) nuclear reaction^12^, for production of X-rays, and in a variety of other accelerator applications and technologies, where small-size accelerator is needed.

## Electronic supplementary material

Supplementary information
