# Supplementary material for: Piezoelectric Accelerator
Source: Sci Rep. 2018 Nov 7;8:16488. doi: 10.1038/s41598-018-34831-8 (PMC6220197; doi:10.1038/s41598-018-34831-8)
Supplement: Supplementary file 1 — Supplementary information [file 41598_2018_34831_MOESM1_ESM.doc]

**Supplementary info for manuscript:**

O.O. Ivashchuk, A.V. Shchagin, A.S. Kubankin, I.S. Nikulin, A.N. Oleinik, V.S. Miroshnik, V.I. Volkov. Piezoelectric accelerator.

**About the experimental setup**

The photography of the experimental setup is shown in the Supplementary Fig. S1.


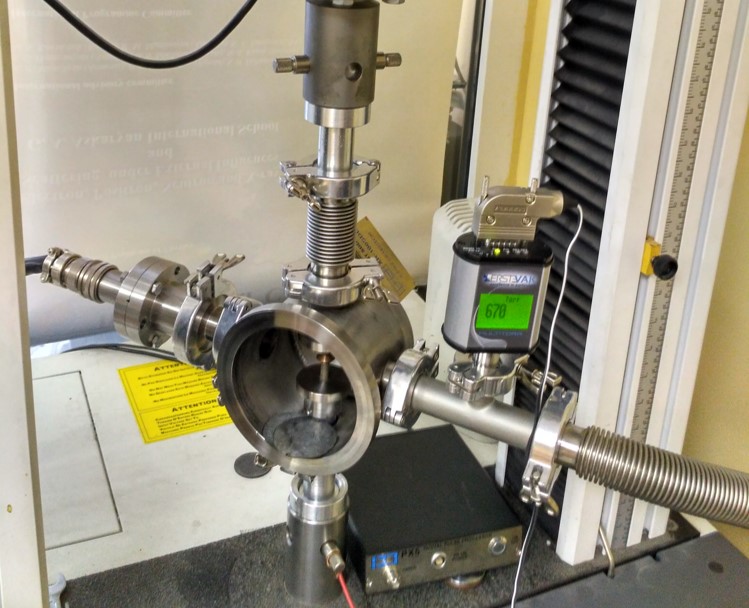


Supplementary Fig. S1. The experimental setup.

The vacuum chamber with the piezoelectric accelerator is installed in the dual column load frame machine Instron 3369. The X-ray detector is installed in the center of the back lid of the chamber. The detector operates with the digital pulse processor (the black box below of the chamber). The front lid of the chamber is removed to show the piezoelectric accelerator. The load machine provides controlled force applied alone the assembly axis at the pressure of the residual gas in the chamber about 0.1 mTorr.


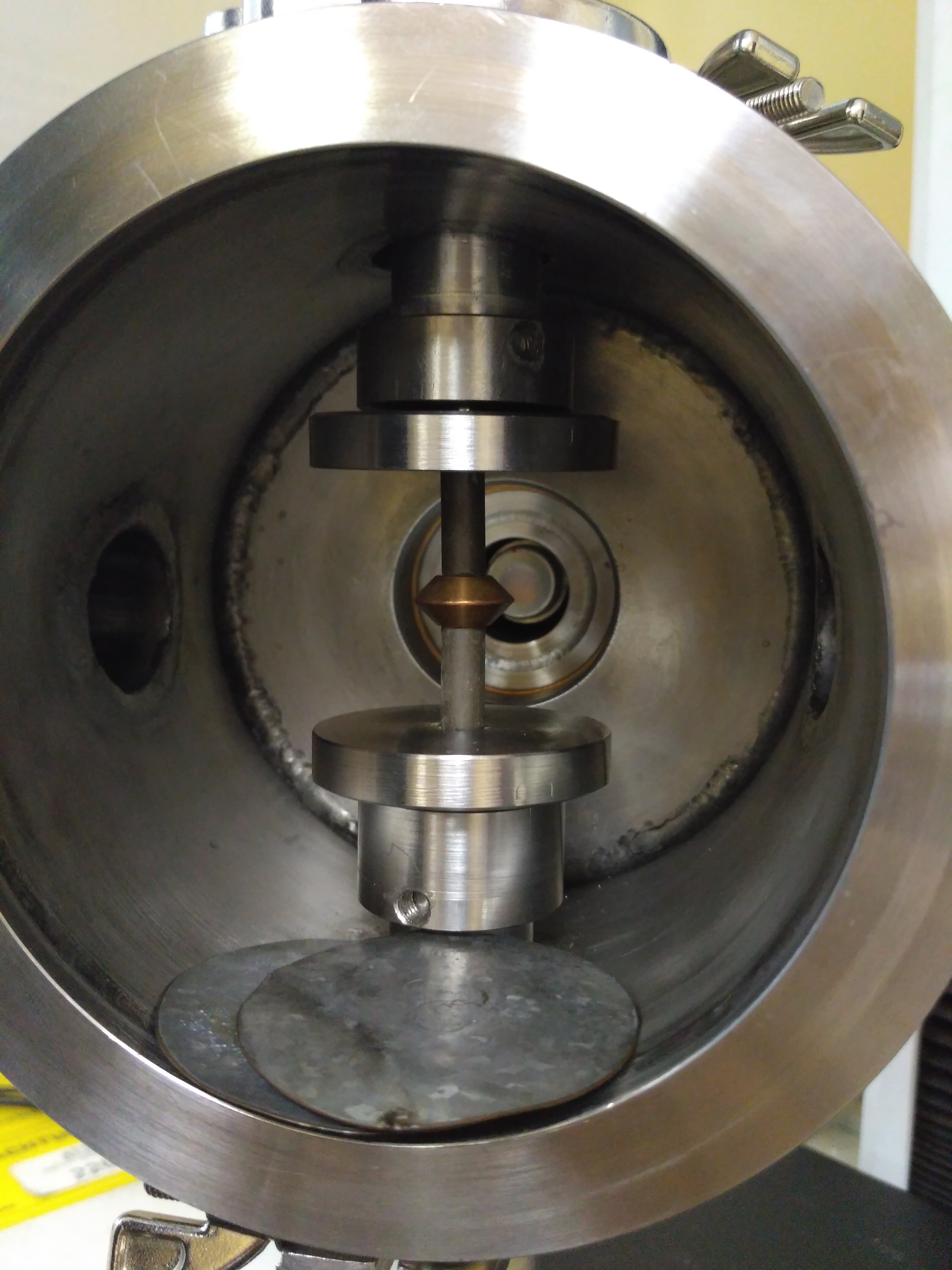


Supplementary Fig. S2. The inner view of the vacuum chamber.

The piezoelectric accelerator in the vacuum chamber is shown in Supplementary Fig. S2. The piezoelectric accelerator consists of the assembly of two piezoelectric ceramic cylinders and the cupper high-voltage electrode. The copper high-voltage electrode is located in the center of the chamber between the ceramic cylinders. The assembly is installed vertically between two metal disks. One can see the entrance window of the X-ray detector with the mylar foil in the center of the back lid of the chamber. Two zinc-plated iron disks are removed and lie in the bottom of the chamber.

**About the ceramic cylinders**

Properties of the ceramic cylinders, according to the producer’s specification:

Piezoelectric modulus, $d_{33}\cdot10^{-12}$ , no less than 170 C/N.

Relative permittivity, $\varepsilon=\varepsilon_{33}^{T}/\varepsilon_{a}$, 600-1300.

Static capacitance 10 – 24.5 pF.

Limit of mechanical strength $P_{\max}$of the piezoelectric element at static compression, no less than 335*10^6^ N/m^2^.

**Data availability statement**

The both measured spectra of X-ray radiation in the .mca format are available upon request.
